# Autoimmunity in Autologous Islet Transplantation

**DOI:** 10.21926/obm.transplant.1803014

**Published:** 2018-07-03

**Authors:** Khawla F. Ali, Vicente T. San Martin, Tyler Stevens, R. Matthew Walsh, Rita Bottino, Massimo Trucco, Betul Hatipoglu

**Affiliations:** 1. Endocrinology and Metabolism Institute, Cleveland Clinic, Cleveland, USA; 2. Digestive Disease Institute, Cleveland Clinic, Cleveland, USA; 3. Institute for Cellular Therapeutics, Allegheny-Singer Research Institute, Pittsburgh, PA, USA

**Keywords:** Total pancreatectomy, autologous islet transplantation, autoimmunity, beta cell failure

## Abstract

Total pancreatectomy (TP) is increasingly being utilized for definitive treatment in patients with debilitating chronic pancreatitis (CP). In an effort to prevent surgical diabetes, the procedure can be performed in conjunction with transplantation of islets of Langerhans recovered from the patients’ own resected pancreas (autologous islet transplantation, AIT). Given that patients undergoing TP and AIT are traditionally assumed not to be at risk for the development of beta-cell autoimmunity, it is possible that the presence of autoimmune islet graft failure has been overlooked and underreported in this patient population. Herein, we describe two cases who underwent TP and AIT and later developed new-onset beta-cell autoimmunity (as evidenced by de novo glutamic acid decarboxylase antibody positivity), accompanied by complete insulin-dependent states. These cases emphasize the need for considering a possible autoimmune phenomenon in the workup of TP and AIT patients who manifest with unexpected and rapid deterioration in their glycemic control.

## Graphical Abstract

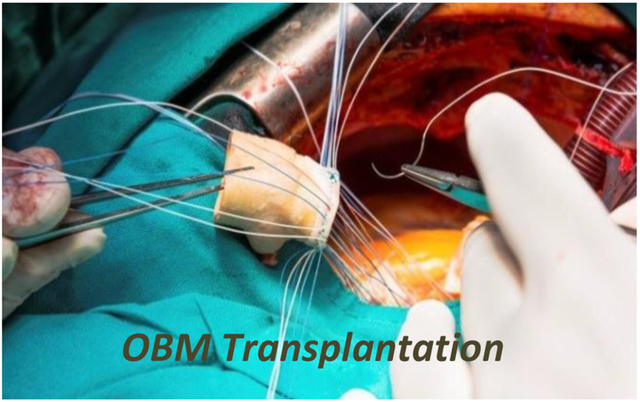

## 1. Introduction

Chronic pancreatitis (CP) is a debilitating disease that can lead to varying degrees of pancreatic endocrine and exocrine dysfunction. One of the most challenging implications of CP is severe abdominal pain, which responds poorly to medical management, and often requires surgical therapy [1]. Total pancreatectomy (TP) is considered the therapy of last resort in patients with debilitating CP, often leading to reduced analgesic use, decreased frequency of hospital admissions for pain, and improvement in quality of life [2]. This surgical procedure can be performed with transplantation of islets of Langerhans (autologous islet transplantation, AIT) recovered from the resected pancreas, and is intended to prevent or attenuate surgical diabetes in select patients based on pre-surgical metabolic assessments [3-5]. AIT appears not to be significantly affected by the stress of cellular rejection encountered in allotransplantation for type 1 diabetes mellitus (DM) [6]. However, development of beta-cell autoimmunity after AIT and subsequent beta-cell rejection have been recently recognized in a report by Bellin et al. [6]. Herein, we report two additional cases of patients with CP, who had no prior history of DM or autoimmunity, both of whom developed new-onset beta-cell autoimmunity and subsequent failure over 12-months post-total pancreatectomy with autologous islet transplantation (TP-AIT).

## 2. Case Presentation

### 2.1 Case 1

A 26-year-old man with history of CP due to R117H heterozygous mutation in the cystic fibrosis gene was seen at our institution in 2013 for TP-AIT. He reported a history of recurrent bouts of acute pancreatitis since 2012, leading to chronic narcotics dependence and malnutrition necessitating tube feeding. He reported no prior history of DM or elevated blood sugars. Additionally, autoimmune work up at the time of evaluation included anti-cyclic citrullinated peptide antibodies (ab) and rheumatoid factor titers both of which were negative. Family history was negative for DM and autoimmune diseases.

**Figure 1:**
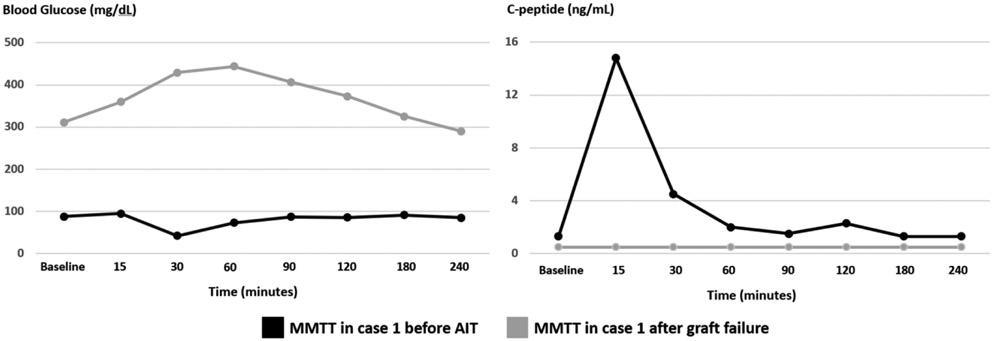
Blood glucose (mg/dL) and C-peptide (ng/mL) trends in case 1 during MMTT completed before AIT (black line) and after islet graft failure (grey line). MMTT: mixed meal tolerance test, AIT: autologous islet transplantation.

On pre-surgical evaluation, his weight and BMI were 80 kg and 22 kg/m2, respectively. Hemoglobin A1c (HbA1c) was 5.0% (reference range: 4.3-5.6%). Initial metabolic testing via mixed meal tolerance testing (MMTT) revealed normal fasting blood glucose (BG) of 88 mg/dL (reference range: 65-100 mg/dL) and normal fasting C-peptide of 1.3 ng/mL (reference range: 0.8-3.2 ng/mL). After the administration of 260 ml of Ensure Plus^®^, his BG and C-peptide peak values were 95 mg/dL and 14.5 ng/mL, respectively (Figure 1). As part of the pre-surgical evaluation protocol, markers for islet cell autoimmunity were obtained; glutamic acid decarboxylase (GAD), insulin, and islet cell abs were negative [GAD ab <5.0 IU/mL (reference range: <5.0 IU/mL), insulin ab <0.4 U/mL (reference range: <0.4 U/mL), and islet cell ab <1:4 (reference range: <1:4)].

Upon successful completion of a comprehensive, multidisciplinary evaluation by our transplantation team (consistent of hepatobiliary surgery, endocrinology, gastroenterology, diabetes education, nutrition, psychology, and pain management), the patient was deemed eligible for TP-AIT. He underwent a successful TP-AIT in December 2013, receiving a total of 598,500 islet equivalent (IEQ), or 7,481 IEQ/kg of body weight. Islets were transfused into the splenic vein with no peri-operative complications. Post-operatively, he was placed on intravenous insulin and glucose infusions and was then transitioned to multiple daily injections of insulin when reliable oral intake had been established. He was discharged on a total daily dose of 16 units of insulin in basal and prandial form. Six months post-discharge, his documented fasting and pre-prandial BGs were in 120-200 mg/dL range, HbA1c was 7.6%, and his insulin dose was adjusted to 16-20 units of insulin per day. Routine re-testing for GAD, insulin and islet cell abs remained negative.

**Figure 2:**
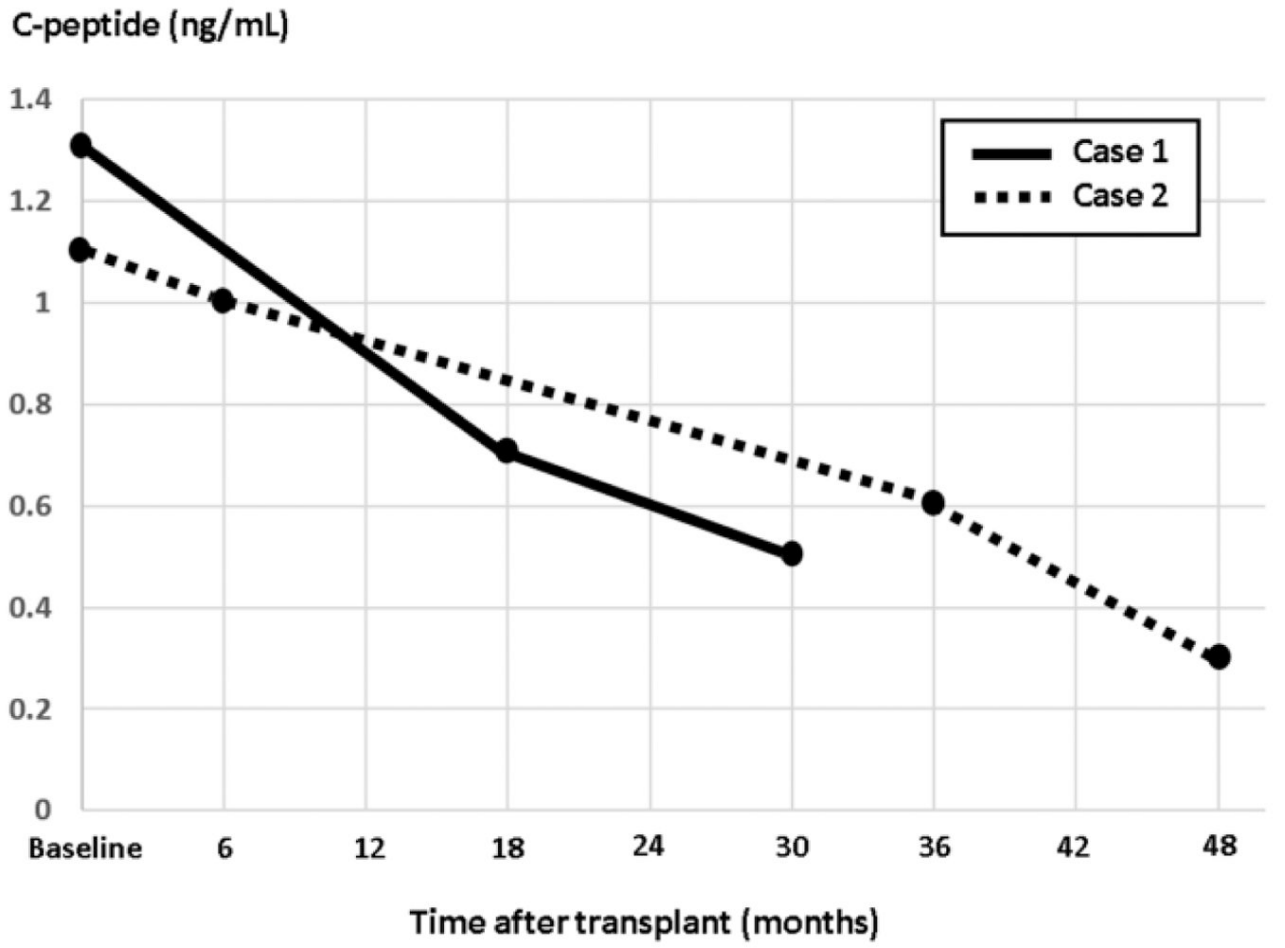
Fasting C-peptide (ng/mL) trends in case 1 (solid line) and case 2 (dotted line) before and after autologous islet transplantation.

The patient subsequently retuned to clinic a few months later reporting unexpected worsening in fasting and pre-prandial BG values ranging from 190-300 mg/dL. He denied any preceding viral illnesses or change in medications. HbA1c checked at the time was 9.5%. Repeat MMTT testing revealed elevated fasting BG of 311 mg/dL and reduced fasting C-peptide of 0.5 ng/mL. Peak BG and C-peptide after administration of 260 ml of Ensure Plus^®^ were 444 mg/dL and 0.5 ng/mL, respectively (Figure 1). Repeat ab testing showed significant elevation in GAD ab titers (>250 IU/mL). Other ab testing remained negative. In alignment with the observed graft failure, patient’s insulin dose was aggressively titrated to achieve adequate glycemic control. Figure 2 shows a graphical presentation of the longitudinal course of fasting C-peptide levels before and after transplantation.

### 2.2 Case 2

A 40-year-old woman with a 9-year history of idiopathic CP presented in 2013 for TP-AIT. Her past medical history was significant for recurrent bouts of acute pancreatitis refractory to medical interventions, resulting in chronic opioid dependence. Work up for pancreatitis included autoimmune markers such as IgG subclasses (1-4) which were negative. Family history was negative for DM or autoimmune disease.

**Figure 3:**
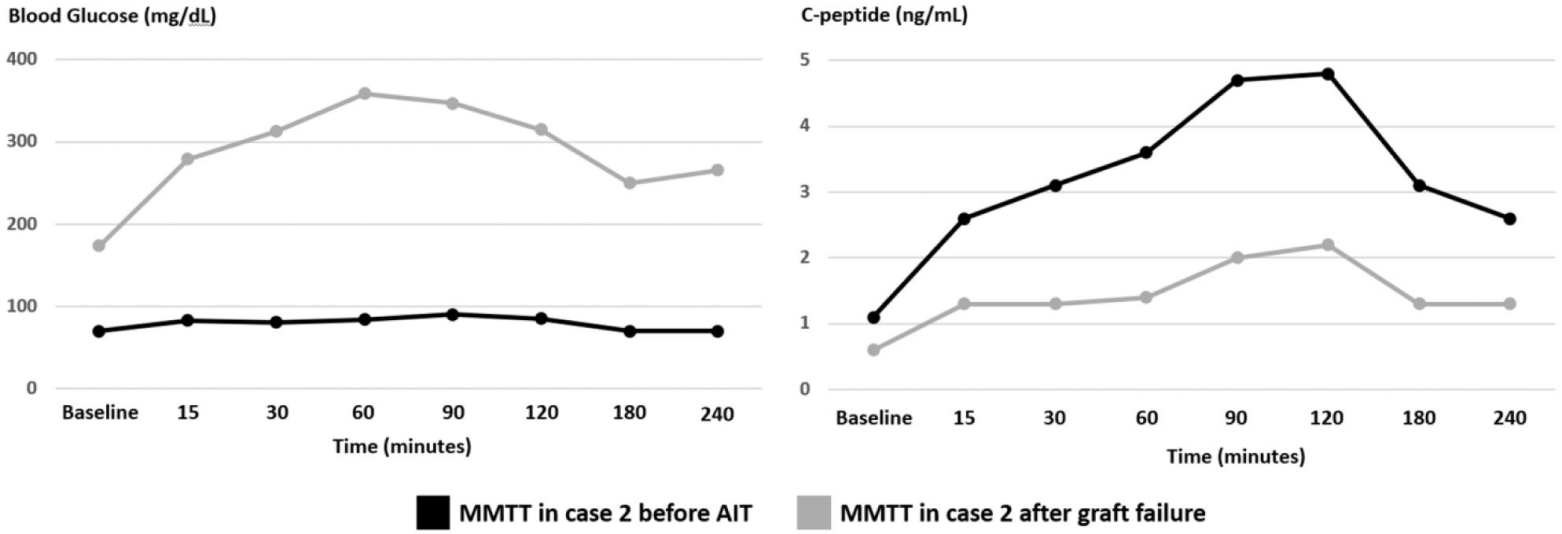
Blood glucose (mg/dL) and C-peptide (ng/mL) trends in case 2 during MMTT completed before AIT (black line) and after islet graft failure (grey line). MMTT: mixed meal tolerance test, AIT: autologous islet transplantation.

On pre-surgical evaluation, weight and BMI were 72 kg and 25.8 kg/m2, respectively. HbA1c was 5.5%. Fasting BG and C-peptide were 70 mg/dL and 1.1 ng/mL, respectively. Post-administration of 360 ml of Ensure Plus^®^, her peak BG was 92 mg/dL and peak C-peptide was 4.8 ng/mL (Figure 3). As part of the pre-surgical evaluation protocol, markers for islet cell autoimmunity were obtained; GAD, insulin, and islet cell abs were all negative.

After deemed eligible for the procedure, based on the metabolic testing above and a comprehensive pre-surgical risk assessment (as outlined in case 1), patient underwent a successful TP-AIT. She received a total of 629,300 IEQ, or 8620 IEQ/kg of body weight, transfused through the splenic vein. She was placed on continuous intravenous insulin and glucose infusions post-operatively and was weaned off insulin within the next few days. She continued to maintain her BG in the range between 70-140 mg/dL off insulin. The patient was discharged on no standing doses of insulin therapy. The patient reported good glycemic control up to 18 months post-transplant. HbA1c during this time period ranged from 6.1-6.3%, and testing for GAD, insulin, and islet cell abs remained negative. Later on, patient was noted to have elevated BG on home testing. HbA1c at the time was 8.5%. On repeat MMTT, fasting BG, and C-peptide were 174 mg/dL and 0.6 ng/mL, respectively. Peak BG and C-peptide were 359 mg/dL and 2.2 ng/mL, respectively (Figure 3). Repeat GAD ab testing was positive (144.9 IU/mL). Islet and insulin ab testing remained negative. The patient was placed on basal and prandial insulin with subsequent adequate glycemic control. Figure 2 shows a graphical presentation of the longitudinal course of fasting C-peptide levels before and after transplantation.

## 3. Discussion

A preponderance of data supports the use of islet transplantation to reverse or prevent diabetes [4, 7-9]. Allogeneic islet transplantation is a well-established therapeutic treatment for a subset of patients with complicated type 1 DM [7], while AIT has become an increasingly recognized approach to prevent or attenuate surgically induced-DM in patients with CP undergoing TP [3, 4].

The outcomes of diabetes-free survival differ across patients that undergo TP-AIT. Analysis of data from our own Cleveland Clinic experience showed that among 36 patients who underwent TP-AIT, and who were insulin independent prior to the procedure, about one third remained insulin independent, one third showed partial islet graft function (requiring some exogenous insulin), and one third became fully insulin-dependent, at a median follow-up of 28-months post-transplantation [3]. These findings were similar to those reported by Sutherland et al., based on data from the largest cohort of TP-AIT to-date [4].

It has been shown that a key predictor of success or failure of autologous islet transplantation is the mass of islets infused, often expressed as total islet equivalent (IEQ) or islet equivalent per kg of body weight (IEQ/kg) [3, 4]. In the study by Sutherland et al., the insulin-independence rate at 3 years was 12% in patients who had received <2,500 IEQ/kg versus 72% in patients who had received >5,000 IEQ/kg [4]. Their findings clearly demonstrate the importance of the mass of islets infused for the post-transplant outcomes, with a higher mass being predictive of superior beta-cell function after TP-AIT. Both our patients had received >5000 IEQ/kg, hence, the acute deterioration in islet cell function was unusual and unexpected.

Other less recognized factors may also affect the outcomes after TP-AIT. For instance, the activation of the innate immune system, with its subsequent inflammatory consequences, is thought to have a detrimental effect on the survival of islets after transplantation. It has been reported that pro-coagulatory and pro-inflammatory cascades are activated within minutes after autologous islet infusion into the liver [10]. The levels of inflammatory cytokines, such as IL-1Ra, IL-6, IL-8 and IL-10, significantly increase within the first 6 hours of islet infusion and gradually subside to pre-transplant levels [10, 11]. In addition to this, experiments in animal models have suggested that the pre-existent inflammatory state associated with hepatic steatosis can also adversely affect the outcomes of AIT [12]. It is therefore conceivable that inflammation imposes some degree of injury to the newly transplanted islet cells, but this would be unlikely to trigger an acute graft failure well over a year after transplantation.

The presence of pre-existent autoantibodies against islet cells has also been linked to worse outcomes after TP-AIT. In a case-series study by Kizilgul et al, insulin dependence was more frequent in patients with elevated GAD ab prior to TP-AIT than in GAD ab negative AIT recipients [13]. More interestingly, the development of de-novo autoantibodies against islet cells after TP-AIT has been associated with acute deterioration of beta-cell function [6]. In the later situation, the infusion of islet cells into a new environment (i.e. the hepatic portal system) may trigger the development of autoantibodies by exposing islet self-antigens to autoreactive T-cells. However, a causative pathogenic role of autoantibodies directed towards beta-cell epitopes is not fully recognized even in type 1 DM. The presence of autoantibodies measured after islet failure is undoubtedly a feature of autoimmunity, but their increased titers may be secondary to release of beta-cell antigen from islet destruction rather than a primary cause of beta-cell failure. Hence, antibody-mediated beta-cell destruction, although a valuable cause of autologous islet graft failure, remains a hypothetical possibility. The mechanisms by which beta-cell damage and autoimmunity is precipitated after TP-AIT are yet to be elucidated.

Finally, given that patients undergoing TP-AIT are traditionally assumed not to be at risk for autoimmune graft failure, it is possible that this rare phenomenon has been overlooked and underreported in the medical literature. In our institution, we have been routinely assessing markers for islet cell autoimmunity prior to and after TP-AIT as part of the baseline panel of tests. Based on the described cases, repeating an autoimmune work-up after TP-AIT is very reasonable and should be considered in patients who manifest an unusually rapid deterioration in their islet cell function, especially when this leads to a complete insulin-dependent state.

## 4. Conclusion

Although patients undergoing TP-AIT have been considered not to be at risk for autoimmune islet cell destruction, autoimmunity should be tested in cases of sudden or unexpected loss in beta-cell function.
